# Predicting neurological outcomes following spinal surgery: A machine learning approach using intraoperative neuromonitoring data

**DOI:** 10.1016/j.xnsj.2026.100927

**Published:** 2026-07-06

**Authors:** Tamir Themans, Valerie Ter Wengel, Saskia van der Gaag, Justin Dauwels, Mark van de Ruit, Niels van der Gaag

**Affiliations:** a Department of Biomechanical Engineering, Delft University of Technology, Delft, The Netherlands; b Department of Neurosurgery, Haaglanden Medical Centre, The Hague, The Netherlands; c Department of Neurology, Haga Teaching Hospital, The Hague, The Netherlands; d Department of Microelectronics, Delft University of Technology, Delft, The Netherlands; e Department of Neurosurgery, Haga Teaching Hospital, The Hague, and Leiden University Medical Center, Leiden, The Netherlands

**Keywords:** Intraoperative neuromonitoring (IONM), Spinal surgery, Motor evoked potentials (MEP), Somatosensory evoked potentials (SSEP), Machine learning (ML), Neurological outcome prediction, XGBoost, Feature importance

## Abstract

**Background:**

Intraoperative neuromonitoring (IONM) reduces postoperative neurological complications, but its precise value for neurological outcomes remains unclear. Machine learning offers a fast, objective, real-time approach to analyzing large IONM datasets. We developed Machine learning models combining baseline characteristics and IONM data—using motor evoked potentials (MEPs) and somatosensory evoked potentials (SSEPs), separately and combined—to predict postoperative neurological outcomes and identify key predictive features.

**Methods:**

In this retrospective cohort study, 67 patients undergoing spinal surgery (2019–2023) at Haga Teaching Hospital with sufficient IONM and clinical data were analyzed. Medical records and 260 IONM features were assessed; neurological status at 3 months postoperatively was categorized in classes relative to preoperative status as “stable deficits”, “intact”, or “improvement”. Using nested cross-validation, 4 classifiers—support vector machine, K-nearest neighbors, random forest, and extreme gradient boosting—were tested in addition to clinical data across MEP, SSEP, and combined modalities. Performance was expressed as sensitivity, specificity, accuracy, and precision.

**Results:**

Extreme gradient boosting outperformed all classifiers on every metric. The combined MEP–SSEP model achieved the highest sensitivity (70.4%), specificity (88.3%), accuracy (87.1%), and best per-class scores, while the MEP model achieved the highest precision (75.6%). Key predictive features were preoperative neurological deficits (29%) and last intraoperative signal latency versus baseline (13.5%).

**Conclusions:**

MEP and SSEP IONM features enhance prediction of 3-months neurological outcomes, provided preoperative status is accurately documented and incorporated. MEP features show superior predictive values compared to SSEP features when both modalities are accessible, with intraoperative signal latency change emerging as a prominent predictive IONM feature.

## Introduction

During spinal surgery for intra- or extramedullary tumors, deformations, or degenerative diseases, surgeons operate in the proximity of critical neural structures. This causes a risk of iatrogenic damage, leading to new or progressive postoperative neurological impairment [1]. To reduce this risk, intraoperative neuromonitoring (IONM) was introduced in 1975 by Tamaki and Yamane [2]. IONM includes measuring several physiological signals, including somatosensory evoked potentials (SSEPs) and motor evoked potentials (MEPs). These signals serve to assess neural pathway integrity and detect real-time changes during spinal surgery [[3], [4], [5], [6], [7]], allowing for a procedural stop or temporary pause to prevent potential damage [1]. The use of IONM has been linked to a decreased occurrence of postoperative neurological complications [4,[8], [9], [10]].

However, IONM requires specialized equipment, trained personnel, and additional time and resources during surgical procedures, leading to increased costs. Furthermore, the successful application of IONM generally relies on the expertise of a trained specialist. Different professionals may interpret signals subjectively, leading to inter-rater variability in predicting neurological outcomes [7,11]. Moreover, in current practice, where IONM is used, no uniform agreements on warning criteria are described [1,6,9,[12], [13], [14], [15], [16], [17]].

Enhanced and objective criteria about the relation between the interpretation of IONM signals and the patient’s prognosis could significantly assist surgeons in making intraoperative decisions. Hence, the ability to real-time predict prognosis would be advantageous. Machine learning (ML) techniques might offer valuable contributions towards achieving this goal [18,19].

Using ML techniques, the excessive amount of IONM data can be rapidly analyzed and interpreted intraoperatively to make predictions about the occurrence of neurological deficits and their key predicting features. A real-time predicting model could serve as an intraoperative decision support system for surgeons, which could help assess the risk of neurological deficits during surgery and aid in making informed choices. Only a few articles have used ML in conjunction with IONM to predict specific outcomes [[20], [21], [22], [23]].

In this study, we investigate the potential of ML techniques to analyze intraoperative IONM data combined with baseline patient data to predict postoperative neurological outcomes following spinal surgery. Key prognostic features will be identified. The goal is therefore to develop an ML algorithm that can effectively predict neurological outcomes after spinal surgery using IONM data, which include both MEPs and SSEPs.

## Methods

A retrospective analysis was conducted of a cohort of patients who underwent spinal surgery monitored with IONM at Haga Teaching Hospital in the Netherlands between October 2019 and August 2023. Patients were included if they underwent surgery for spinal tumors, scoliosis correction, kyphosis correction, untethering procedures, tumor biopsies, vertebral fusion, decompression, corpectomy, or cordotomy. IONM was primarily applied in more complex spinal procedures, such as those listed above, and is not routinely used in low- to moderate-complexity cases, which contributes to a relatively limited cohort size. Patients without sufficient IONM data or without sufficient pre- and postoperative neurological status were excluded.

**Fig. 1:**
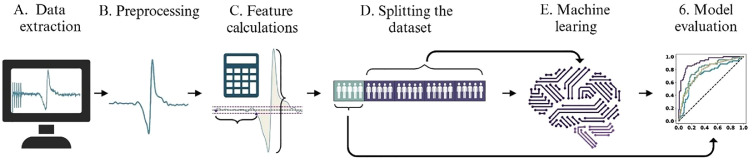
Schematic representation of the used methodology to conduct this study. (A) Data extraction of intraoperative neuromonitoring and additional features, (B) signal preprocessing to enable feature calculations, (C) feature calculations, (D) splitting the dataset in 80% training set and 20% testing set, (E) machine learning algorithm trains on the training set, (F) model evaluation based on predictions made by the machine learning algorithm and actual outcomes from the test-set. Created with BioRender.com.

Neurological status was assessed during patient consultations, including preoperatively, postoperatively, and at defined follow-up moments, using standardized neurological assessments. Baseline neurophysiological data, including MEPs and SSEPs, were obtained at the start of each surgery. The study was approved by the Medical Ethics Assessment Committee of Leiden-Den Haag-Delft, and due to its retrospective nature, informed consent was not obtained from the patients. For an overview of the steps taken in the methodology of this study, see Fig. 1.

### Data extraction

Various types of data needed to be collected to train the ML algorithm, including IONM features, additional patient-related features, and the neurological status 3 months postoperatively compared to the preoperative status. The latter is described as the outcome value and was categorized into 3 classes: “Neurologically stable deficits”, “Neurologically intact”, and “Neurological improvement”. “Neurologically stable deficits” encompass uni or bilateral paresis or paralysis and/or any sensory deficits compared to baseline. “Neurological improvement” was defined as any improvement in neurological status compared to baseline. “Neurologically intact” was defined as no change in neurological status compared to baseline.

Additional patient features and the outcome value were retrospectively extracted from the electronic health record. Lastly, IONM signals were retrieved from Medtronic’s NIM-Eclipse, a system used for generating, recording, and storing IONM data during the surgical procedures.

### Specific data extraction IONM

Depending on the patient’s characteristics and surgical procedure, different muscle groups were monitored intraoperatively. The routinely and consistently measured muscle groups across the included patients, and therefore incorporated into the present analysis, were the tibialis anterior (TA), the abductor hallucis (AH), and the Gastrocnemius muscle. The reference electrode was positioned on the abductor digiti minimi (ADM). During surgeries involving regions above cervical-8 (C8), the ADM was also measured routinely. Thus, these 4 muscle groups, measured bilaterally, correspond to 8 measured muscle groups per patient. To activate these muscle groups, a corkscrew electrode is bilaterally positioned on the patient’s motor cortex. A pulse train stimulus of 4 to 5 pulses within the range of 150 to 650 volts was applied to generate an MEP signal, while needle electrodes were placed in the measured muscles to monitor the measured muscle responses.

Regarding the SSEP signals, different tracts labeled under distinct names were documented for each patient. However, each patient presented 1 or more signals representing the left leg, as well as the right leg. The decision was made to incorporate, from each patient, 1 corresponding tract for both the left and right leg, resulting in 2 SSEP pathways per patient. In patients where multiple signals per lower extremity were measured, the signal with the largest feature value was included. To activate the sensory pathways, needle electrodes are inserted into the corresponding nerves, while corkscrew electrodes on the head located above the somatosensory cortex are employed to capture the signals. The intensity was set within the range of 7 to 20 mA. MEP and SSEP signals were captured with a sampling frequency of 10,000 Hz.

### Raw data processing

IONM signals underwent preprocessing in Python 3.8 [24]. Any signals measured prior to the last indicated baseline measure were excluded from the dataset. A fourth-order zero-phase Butterworth low-pass filter was employed with a cutoff frequency of 300 Hz to filter out noise from the signal.

### Feature selection

**Fig. 2:**
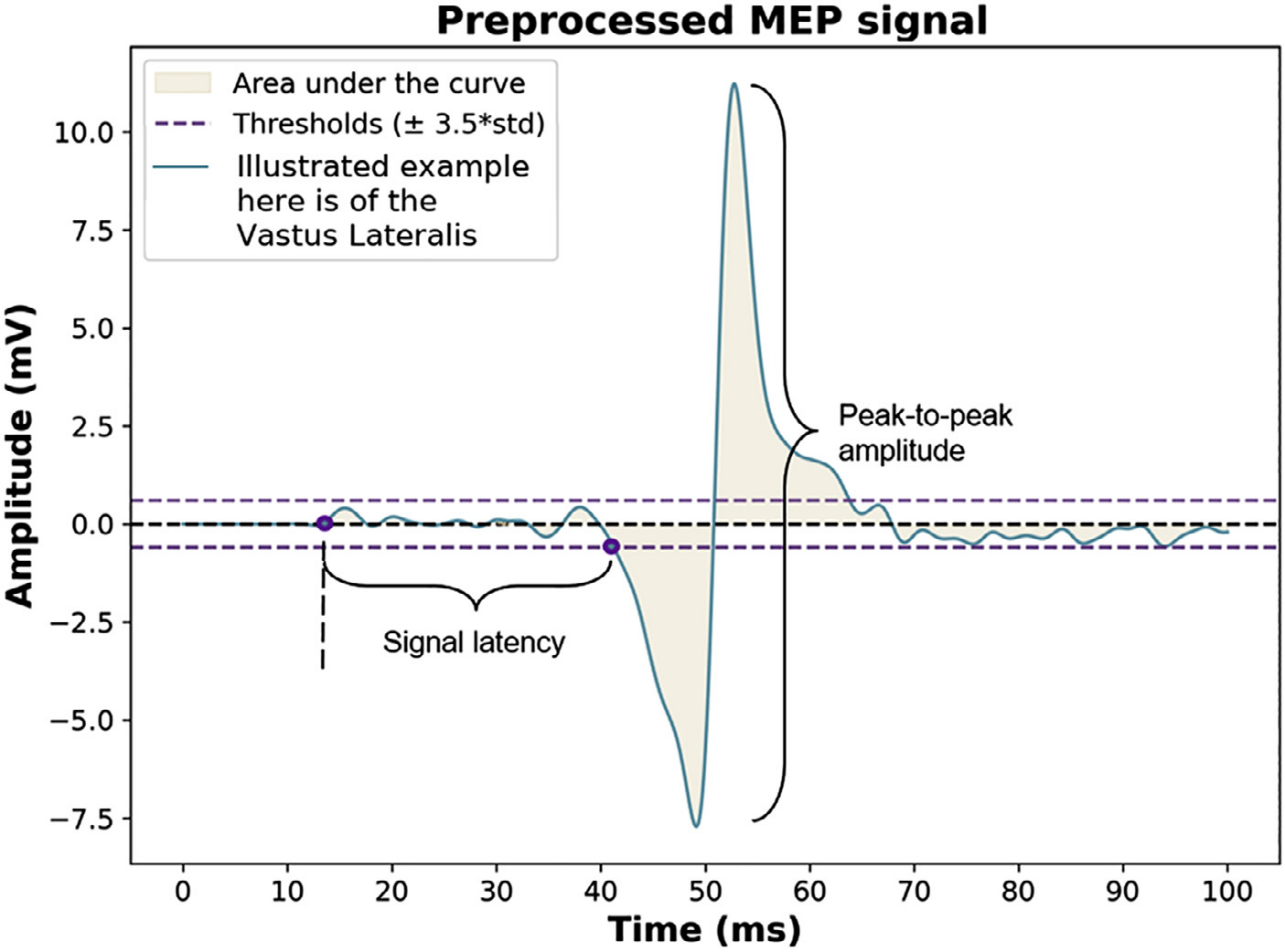
Visualization of the signal properties peak-to-peak amplitude, area under the curve and signal latency. std, standard deviation.

IONM features were calculated based on different signal characteristics, specifically peak-to-peak values, area under the curve (AUC) measurements, and latency parameters (Fig. 2). The latter is included because, similar to peak-to-peak and AUC values, it may contain critical information about the signal’s flow dynamics, and thus potentially, one’s postoperative neurological status [23].

In addition, 4 features were considered in this study: the mean intraoperative mean arterial pressure (MAP) [25,26], the presence or absence of preoperative neurological deficits [8,17,[27], [28], [29]], and patient age [18,27]. Also, the surgical location, described as “Above thoracic 12”, or “Below thoracic 12” was considered. This division was made to distinguish between lesions at the spinal cord level and those below it. When the surgical location spans vertebrae both above and below thoracic 12, the area is considered as “Above thoracic 12”.

### Data preprocessing for machine learning

A StandardScaler, which uses the Z-score, was used to scale the data for ML. The not a number (NaN) values were replaced by the median value of the column. Furthermore, in cases where intraoperative blood pressure values were not available (n = 2), the median value of the mean intraoperative MAP, derived from all other patients, was used as a substitute.

### Splitting the dataset

In this study, nested cross validation (CV) is used to split the dataset. Both the outer and the inner CV loops were divided into 5 folds. In this study, nested CV was used to split the dataset to ensure an unbiased estimate of model performance while optimizing hyperparameters. The outer CV loop, responsible for evaluating generalization performance, was divided into 5 folds. Similarly, the inner CV loop, used for hyperparameter tuning, also employed 5 fold CV within each outer fold.

### Model development

Four different ML classifiers were used to construct the models:

• Support vector machine (SVM).
• K-nearest neighbors (KNN).
• Random forest (RF).
• Extreme gradient boosting (XGBoost).

These classifiers were chosen to cover a diverse range of algorithmic approaches, including linear and nonlinear methods, ensemble techniques, and models known for their performance in medical datasets [23,28,30]. This selection allows for a broad comparison of model performance and provides insight into their applicability to the dataset in question.

#### Support vector machine (SVM)

SVM aims to find the optimal hyperplane that best separates data points into different classes by maximizing the margin between them [31]. It is effective in high-dimensional spaces, making it suitable for our dataset [32].

#### K-nearest neighbors (KNN)

KNN classifies new data points based on the majority class of their nearest neighbors, with “k” determining how many neighbors are considered. It is computationally efficient for small datasets and was chosen due to its strong performance in previous related studies [23]. KNN can struggle in scenarios with overlapping classes, which is relevant in our case involving a 3-class classification problem, as class boundaries may not be well-defined [33].

#### Random forest (RF)

RF is an ensemble method that combines multiple decision trees, each trained on randomly selected data, to improve prediction accuracy [34]. It handles high-dimensional data well [35] and allows for easy computation of feature importance.

#### Extreme gradient boosting (XGBoost)

XGBoost is a gradient boosting method that builds an ensemble of decision trees iteratively. Unlike RF, it adds new trees successively, while taking into account the errors of previous trees [36].

## Results

### Data

Initially, data were obtained from 69 patients who underwent surgery between October 2019 and August 2023 (median age was 58, standard deviation 16.6, and range 19–87). Of these patients, 48 showed preoperative neurological deficits such as loss of strength or hypesthesia, while the remaining 21 reported no preoperative neurological deficits. Three months postoperative, 39 patients exhibited neurological improvement, 20 were neurologically intact, and 10 showed neurologically stable deficits, of whom 3 within this last group experienced slight neurological worsening.

**Fig. 3:**
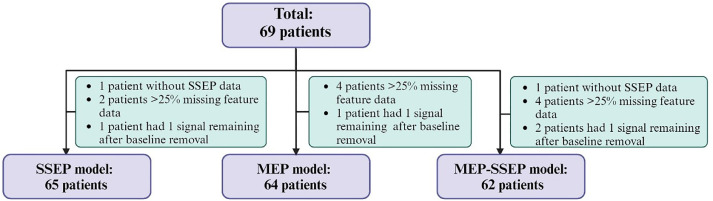
Flowchart of the patients included for each modality with their exclusion criteria. MEP, motor evoked potential; SSEP, somatosensory evoked potential. Created with BioRender.com.

After patients with insufficient data were removed, the SSEP model included 65 patients, the MEP model included 64 patients, and the MEP-SSEP model included 62 patients (see Fig. 3). In total, 67 patients were included in one of the 3 ML models (see Table 1). For a detailed list of individual patient data, see “Appendix A”.

**Table 1:** Summary of patient characteristics of all 67 patients included in at least one of the 3 machine learning models.

Characteristic	Value
Age (y)	Median: 58.0 (range 19–87)
Pathology	Tumor: 47 (70.1%), deformity: 7 (10.4%), degenerative: 8 (11.9%), and other: 5 (7.5%)
Level of pathology	Thoracic 12 or above: 39 (58.2%) and below thoracic 12: 28 (41.8%)
Preoperative neurological deficit	Yes: 44 (65.7%) and no: 23 (34.3%)
Mean intraoperative MAP	Mean: 83.5 mmHg (SD ± 11.8)
Neurological outcome	Improvement: 41 (61.2%), intact: 21 (31.3%), and stable deficits: 5 (7.5%)

MAP, mean arterial pressure.

In total, 260 features were identified per patient. A complete overview of the calculated features is presented in Table 2.

**Table 2:** Calculation of the 256 IONM features.

Feature origin	Feature calculation description	Modality	
		Eight MEP muscles	Two SSEP tracts
Peak-to-peak:	Maximum amplitude drop of a signal compared to the preceding signal	✓	✓
Peak-to-peak:	Maximum amplitude increment of a signal compared to the preceding signal	✓	✓
Peak-to-peak:	Maximum % amplitude drop of a signal compared to the preceding signal	✓	✓
Peak-to-peak:	Maximum % amplitude increment of a signal compared to the preceding signal	✓	✓
Peak-to-peak:	Maximum amplitude drops of a signal compared to the baseline signal	✓	✓
Peak-to-peak:	Maximum amplitude increment of a signal compared to the baseline signal	✓	✓
Peak-to-peak:	Maximum % amplitude drop of a signal compared to the baseline signal	✓	✓
Peak-to-peak:	Maximum % amplitude increment of a signal compared to the baseline signal	✓	✓
Peak-to-peak:	Amplitude difference of the last measured signal compared to the baseline signal	✓	✓
Peak-to-peak:	% amplitude difference of the last measured signal compared to the baseline signal	✓	✓
AUC	Maximum AUC decrease of a signal compared to the preceding signal	✓	✓
AUC	Maximum AUC increment of a signal compared to the preceding signal	✓	✓
AUC	Maximum % AUC decrease of a signal compared to the preceding signal	✓	✓
AUC	Maximum % AUC increment of a signal compared to the preceding signal	✓	✓
AUC	Maximum AUC decrease of a signal compared to the baseline signal	✓	✓
AUC	Maximum AUC increment of a signal compared to the baseline signal	✓	✓
AUC	Maximum % AUC decrease of a signal compared to the baseline signal	✓	✓
AUC	Maximum % AUC increment of a signal compared to the baseline signal	✓	✓
AUC	AUC decrease of the last measured signal compared to the baseline signal	✓	✓
AUC	% collapse of the last measured signal compared to the baseline signal	✓	✓
Latency	Maximum delay of a signal compared to a preceding signal	✓	
Latency	Maximum % delay of a signal compared to a preceding signal	✓	
Latency	Maximum delay of a signal compared to the baseline signal	✓	
Latency	Maximum % delay of a signal compared to the baseline signal	✓	
Latency	Delay of the last measured signal compared to the baseline signal	✓	
Latency	% delay of the last measured signal compared to the baseline signal	✓	
Latency	Longest delay of all signals	✓	

AUC, area under the curve; MEP, motor evoked potential; SSEP, somatosensory evoked potential.

### Overall classifier results

**Fig. 4:**
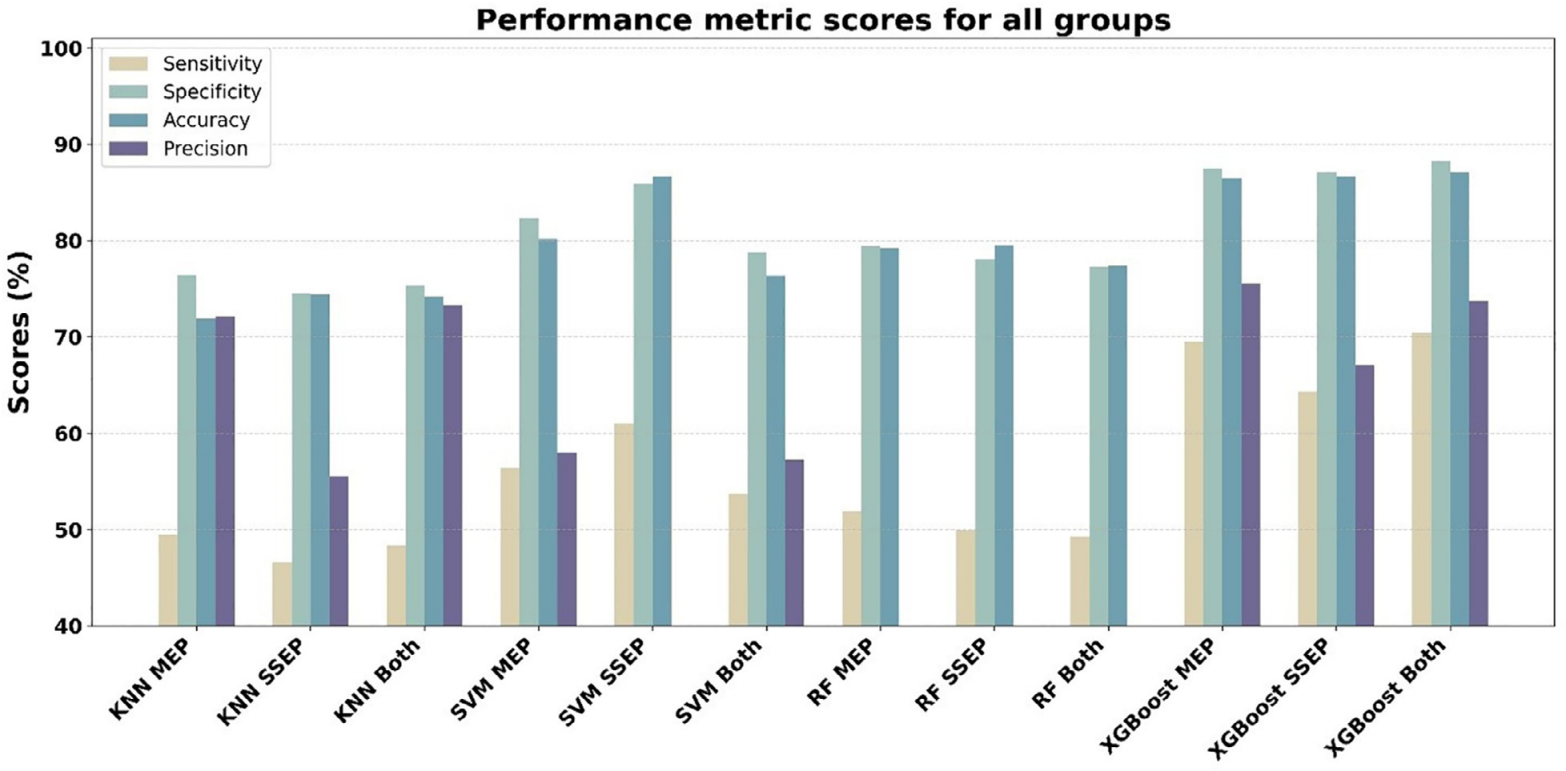
Classifier performance metrics for each modality MEP, SSEP, and both (MEP and SSEP). Scores are percentages and are a combined score over the 3 outcome classes “neurologically stable deficits”, “neurologically intact”, and “neurological improvement”. KNN, K-nearest neighbors; SVM, support vector machine; RF, random forest; XGBoost, extreme gradient boosting. Note: for some models the precision couldn’t be calculated due to division by zero.

Fig. 4 lists all the classifier performances applied to the MEP, SSEP, and combined MEP-SSEP datasets, illustrating that the XGBoost classifier outperformed the other classifiers on all evaluated aspects. Overall performance across the 3 outcome classes for the combined MEP-SSEP model gives XGBoost a sensitivity of 70.4%, specificity of 88.3%, accuracy of 87.1% and precision of 73.7%.

### Individual modalities results

**Fig. 5:**
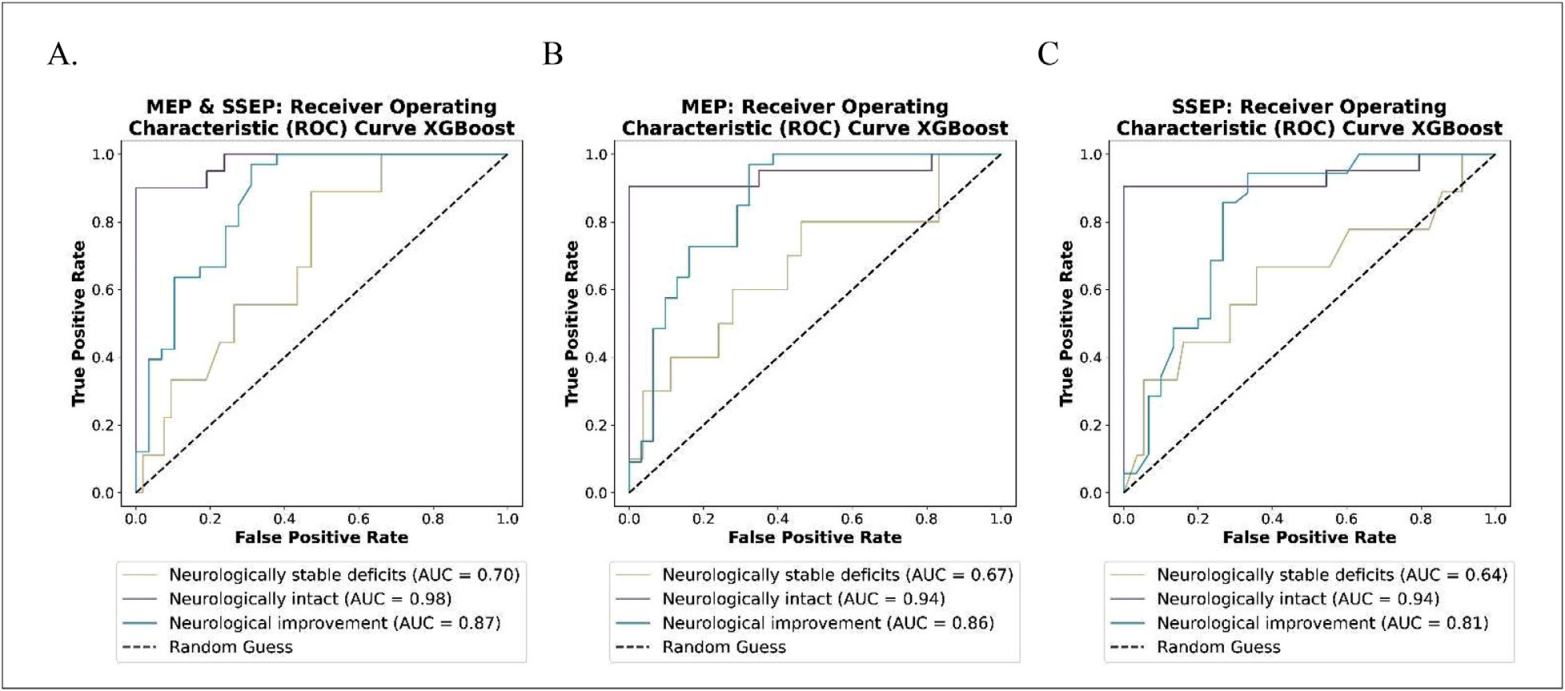
ROC curves per individual outcome class by the XGBoost classifier for all separate models. (A) MEP and SSEP, (B) MEP, and (C) SSEP. MEP, motor evoked potential; SSEP, somatosensory evoked potential; XGBoost, extreme gradient boosting.

**Fig. 6:**
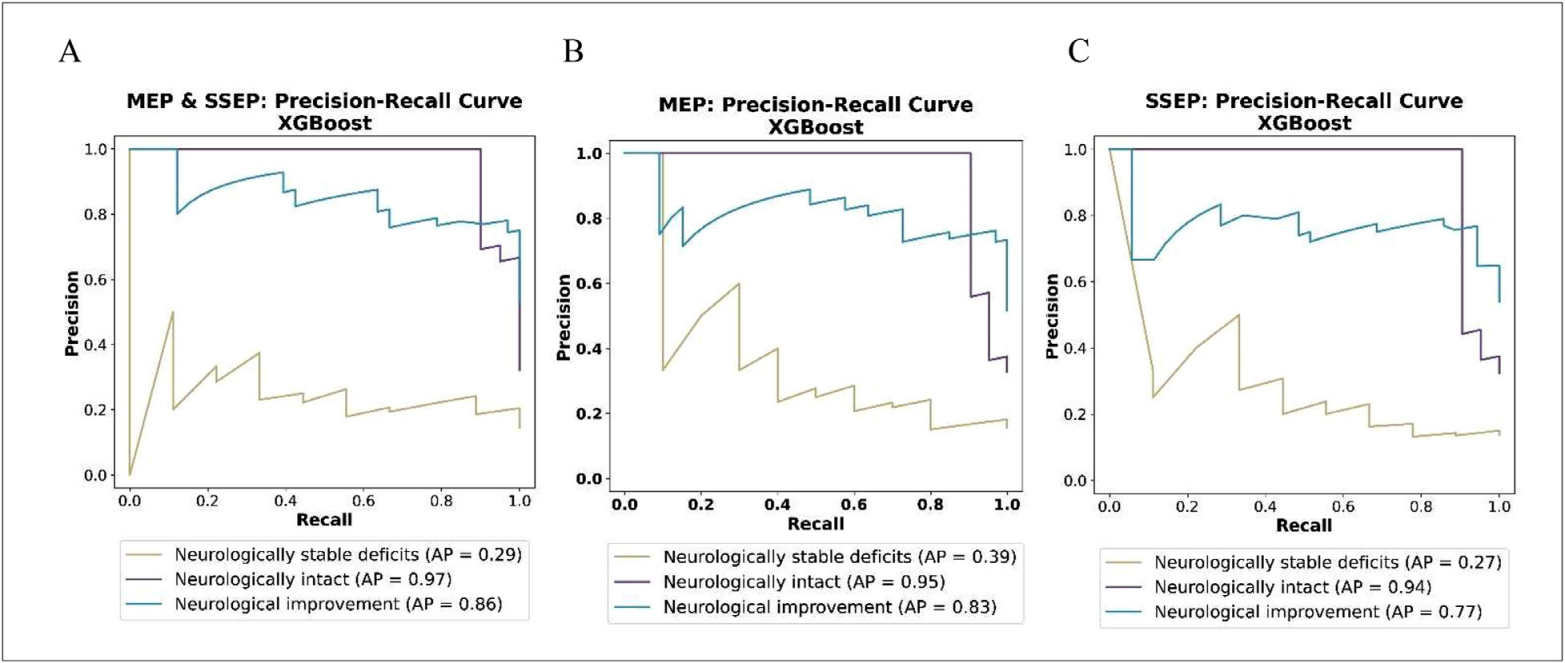
Precision-recall curves per individual outcome class by the XGBoost classifier for all separate models. (A) MEP and SSEP, (B) MEP, and (C) SSEP. MEP, motor evoked potential; SSEP, somatosensory evoked potential; XGBoost, extreme gradient boosting.

Fig. 5 shows receiver operating characteristics (ROC) curves for each individual class, plotted for the combined MEP–SSEP, MEP, and SSEP XGBoost models. The highest scores are observed in the combined MEP–SSEP model, with an AUC of 0.70 for “Neurologically stable deficits”, 0.98 for “Neurologically intact”, and 0.87 for “Neurological improvement”. Fig. 6 shows the respective precision-recall curves for each individual modality for each outcome class. “Neurologically stable deficits” was best predicted by the MEP model (average precision [AP] = 0.39), whereas “Neurologically intact” and “Neurological improvement” were best predicted by the combined MEP–SSEP model, with APs of 0.97 and 0.86 respectively.

**Fig. 7:**
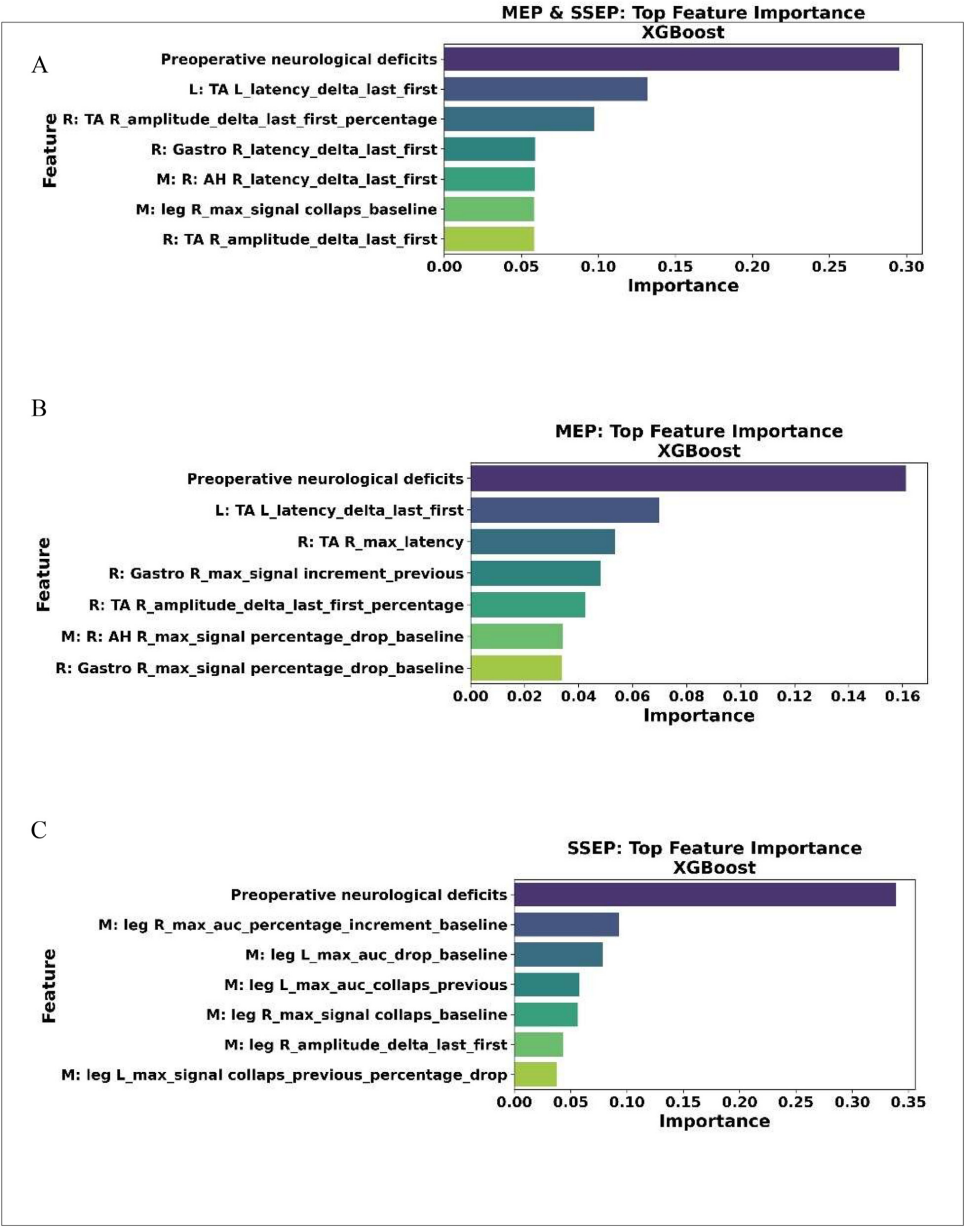
Feature importance plots for the 3 models. The 7 features which hold the highest significance are plotted. (A) MEP and SSEP, (B) MEP, and (C) SSEP. MEP, motor evoked potential; SSEP, somatosensory evoked potential; XGBoost, extreme gradient boosting.

In all 3 models, the presence or absence of preoperative motor or sensory deficits is the most prominent feature, contributing about 29%, 16%, and 33% in the predictions of the MEP–SSEP model, the MEP model and the SSEP model, respectively (see Fig. 7). This is followed by the MEP feature “latency of the last measured TA signal relative to baseline”, contributing 13.5% and 7% for the MEP–SSEP model and the MEP model, respectively. Latency is therefore the signal characteristic with the highest feature importance. The second most predictive feature in the SSEP model is the “percentage AUC increase in a signal from baseline for the right leg”, contributing for 9%. Additional features, such as age, surgical location, and mean intraoperative MAP, were not incorporated in this figure because of their negligible values in these ML models.

## Discussion

In this study, ML was used to predict neurological outcomes 3 months postoperatively, using both IONM characteristics and additional patient-related factors. The aim was to correctly classify patients into 3 groups: “Neurologically stable deficits”, “Neurologically intact”, and “Neurological improvement”, and analyze their key predicting features. Four ML classifiers were constructed, with XGBoost showing superior performance in all performance metrics. Notably, the combined MEP–SSEP model exhibited the highest scores for sensitivity: 70.4%, specificity: 88.3% and accuracy: 87.1%, while the MEP model exhibited the highest performance for precision: 75.6%. Overall, the model shows a high predictive performance for predicting neurological outcomes within the 3 distinct groups.

On average, the combined MEP–SSEP model yields the most favorable outcome scores: AUC = 0.70, 0.98, 0.87, and AP = 0.29, 0.97, 0.86 for “Neurologically stable deficits”, “Neurologically intact”, and “Neurological improvement”, respectively. These findings not only highlight the ability to predict neurological outcomes but also shed light on key predicting features and identify those of less significance.

Only 1 study has examined the use of IONM data to predict neurological outcomes of patients after spinal surgery previously. Jamaludin et al. [23] used solely MEP data to categorize patients into those exhibiting positive functional outcomes and those showing no changes from preoperative to postoperative. Only baseline and final IONM measurements were used, along with a limited number of features. They focused on the outcome measures “Neurological improvement” and “No improvement” when predicting neurological outcomes after spinal surgery using IONM data. Their best-performing model, employing a KNN classifier, showed relatively high sensitivity (87.5%) but low specificity (33.3%). Comparison of these findings with our results shows higher specificity (88.3%) but lower sensitivity (70.4%) in our study. The higher average scores of specificity + sensitivity in our study can be attributed to the integration of a larger set of IONM features along with their specific calculations, the inclusion of additional features, and the targeted training of multiple algorithms using nested CV. Therefore, it is important for future research to implement these applications. Nevertheless, it is important to remember that higher sensitivity is preferred over higher specificity in this type of study. This preference arises from the goal of correctly classifying patients into the positive group (true positives) rather than the correct classification of the negative group (true negatives). Hence, future research should explore methods to enhance sensitivity without excessively compromising specificity.

Previous research indicates a superior predictive value for MEP signals compared to SSEP signals. For example, Antkowiak et al. [37] showed higher sensitivity (92.3%) and specificity (81.8%) for MEPs as opposed to SSEPs (50% sensitivity and 81% specificity), while comparing significant IONM alerts with a patient’s postoperative neurological outcome. Consistent with these trends were studies conducted by Cannizzaro et al. [17] and Dauleac et al. [9]. In our study, the MEP model outperformed the SSEP model slightly with higher sensitivity (69.5% vs. 64.3%), specificity (87.5% vs. 87.1%), and precision (75.6% vs. 67.1%), while the SSEP model performs slightly better on accuracy scores (SSEP: 86.7% vs. MEP: 86.5%). Looking at the specific contributions per individual class predictions, MEP outperformed SSEP slightly on both AUCs and APs. The similarity of these values highlights the importance of including SSEP signals in intraoperative assessment. Despite challenges in interpreting real-time SSEP signals, their individual importance in predicting neurological outcomes 3 months postoperatively is nearly equal to MEP importance scores. However, the feature importance plots show that of the top 7 features in the combined MEP-SSEP model, 5 relate to MEP, while only 1 relates to SSEP. Thus, when both modalities are available, MEP features have greater predictive value.

The presence or absence of preoperative neurological deficits is the most important feature in all 3 models, contributing about 29%, 16%, and 33% in the predictions of the MEP–SSEP, MEP, and SSEP models, respectively. To assess the exact impact of this feature, a sensitivity analysis was performed by removing this feature from the combined MEP–SSEP model (see “Appendix B”). The findings show that the model retains its predictive ability in terms of AUCs, showing 0.71, 0.75, and 0.54 for “Neurologically stable deficits”, “Neurologically intact”, and “Neurological improvement”, respectively. However, a significant performance reduction was observed for the “Neurologically intact” and “Neurological improving” groups. Moreover, the calculated APs indicate unreliable predictions without this characteristic, with scores of 0.26, 0.57 and 0.58 for “Neurologically stable deficits”, “Neurologically intact”, and “Neurological improvement”, respectively. Consequently, the reliability of the model depends on the inclusion of this feature. This is in line with the existing literature [8,17,[27], [28], [29]]. For example, Cannizzaro et al. [17] stated that the most important predictor associated with an improved score on the modified McCormick scale was the patients’ preoperative neurological status (p < 0.001). Similar trends were shown by Merali et al. [27], Wang et al. [8], Zhang et al. [29], and Shimizu et al. [28]. This study can therefore be considered a proof of concept, demonstrating that while IONM-based features clearly have potential value, their predictive value in the current cohort remains secondary to the preoperative clinical status. However, this may improve with larger datasets.

Currently, most physicians rely primarily on signal collapse and signal increment to interpret MEP and SSEP signals intraoperatively. In the best-performing model, 3 out of the top 5 features are latency features. This highlights the importance of including signal latency in the visual intraoperative assessment, in addition to signal collapse and increment. As no clear thresholds for latency changes have yet been established, these features should currently be interpreted alongside amplitude-based criteria and communicated as supplementary information.

Latency changes can be reported in real time to the surgical team alongside amplitude changes as a potential early warning signal, which may support intraoperative decision-making, like pausing or terminating the procedure. The current literature barely addresses the relationship between MEP signal latencies and postoperative neurological outcomes, as amplitude changes seem to have a greater predictive value [38]. Nevertheless, our study shows different results, highlighting the need to further explore the neurological predictive value of latency features. However, these findings should be interpreted with caution. Amplitude-based MEP changes remain the most established warning criteria in current IONM practice, while the clinical significance of latency changes has not yet been clearly established. Latency changes may also be more susceptible to false positive interpretation due to anaesthetic-related effects. The prominence of latency features in our model may therefore partly reflect the relatively small sample size of this cohort. Larger multicenter datasets are required to confirm their independent predictive value.

Age and Surgical location hold an invaluable significance in all 3 models. This suggests that the decision to use IONM should be made irrespective of surgical location or age. However, it should be noted that the surgical location feature is strongly normalized as above or below T12. Moreover, the mean intraoperative MAP does not figure prominently. This contradicts existing literature suggesting that this feature has a high predictive value for postoperative neurological deficits [25]. Our study found neurological improvements both at the highest (135.92 mmHg) and lowest (61.66 mmHg) measured mean intraoperative MAP. This finding can be explained by the fact that only the average intraoperative MAP was included in the analysis. This approach does not prove insight into temporary hemodynamic changes or the clinical response to IONM alerts. For example, MAP is often actively increased following a change in the IONM signal, which means that the mean MAP value may mask relevant periods of temporary hypotension. Therefore, the current feature representation may underestimate the true relationship between changes in MAP, IONM changes, and postoperative neurological outcomes. Future studies should therefore include detailed temporal MAP measurements to better assess the predictive value of intraoperative hemodynamic fluctuations.

Furthermore, all patients included in the study without preoperative deficits had no neurological worsening postoperatively. In contrast, 10 out of the 48 patients with preoperative neurological deficits showed no neurological improvement (20.8%), of whom 3 deteriorated (6.3%). Therefore, for patients who wish to postpone surgery until actual neurological symptoms are experienced, such as some patients with a benign spinal cord tumor, it may be strongly advised to have the surgery before developing these symptoms, because this significantly reduces their chances of maintaining or developing postoperative neurological deficits.

Our study is inevitably accompanied by limitations. Apart from subjectivity and ambiguous alert criteria, several factors contribute to the complexity of interpreting IONM data. Considerations such as the general neurological condition of the patient, suboptimal placement of needle electrodes, and anesthesia generally play an important role in successful interpretation [39]. However, in the present prospective series these features did not have any signs of flawed data in this respect.

In our study, only 4 muscle groups were measured in each patient, facilitating their inclusion in this type of ML, regardless of whether this was an affected muscle or not. It is possible that our model would show greater predictive value of IONM features if the affected muscle was included in the ML model for each patient. However, conducting similar studies with this recommendation presents challenges because this specific muscle group must be measured in each patient individually to train the ML algorithm, which is often not the case in practice.

In addition, we assessed the neurological status 3 months postoperatively. However, neurological outcomes immediately postoperative were not systematically recorded and therefore could not be included in this analysis. Future studies incorporating both immediate and long-term postoperative follow-up assessments may provide a more comprehensive understanding of the temporal relationship between intraoperative IONM changes and neurological recovery.

Lastly, to include a separate group with neurological worsening, more data is needed. Therefore, a larger or multicenter study seems recommended. This would improve the training of the ML algorithm, likely leading to better results. A potential class imbalance due to the small size of the third outcome group (“neurologically stable deficits”, 7.5%), should also be considered. A binary outcome definition may therefore have been more appropriate for this sample size. In addition, the model has only been internally validated using nested cross-validation and not yet tested on an external cohort, limiting its clinical applicability. Future studies should focus on multicenter external validation.

## Conclusion

MEP and SSEP IONM features enhance the prediction of neurological outcomes 3 months postoperatively, provided that the patient’s preoperative status is accurately documented and included in the prediction. Though either MEP or SSEP features alone offer predictive value, MEP features show superior predictive values compared to SSEP features when both modalities are accessible, with latency emerging as a prominent predictive IONM feature. Further research with larger series is recommended to provide more robust predictions.

## CRediT authorship contribution statement

**Tamir Themans:** Conceptualization, Data curation, Formal analysis, Investigation, Methodology, Project administration, Resources, Software, Validation, Visualization, Writing – original draft, Writing – review & editing; **Valerie Ter Wengel:** Conceptualization, Methodology, Supervision, Writing – review & editing; **Saskia van der Gaag:** Data curation, Resources; **Justin Dauwels:** Software, Validation, Writing – review & editing; **Mark van de Ruit:** Conceptualization, Methodology, Resources, Supervision, Validation, Writing – review & editing; **Niels van der Gaag:** Conceptualization, Methodology, Resources, Supervision, Validation, Writing – review & editing.

## Funding

This study received no financial support.

## Declaration of competing interests

The authors declare that they have no known competing financial interests or personal relationships that could have appeared to influence the work reported in this paper.

## Appendix Supplementary materials

Image, application 1
