# Supplementary material for: Predicting neurological outcomes following spinal surgery: A machine learning approach using intraoperative neuromonitoring data
Source: N Am Spine Soc J. 2026 Jul 6;27:100927. doi: 10.1016/j.xnsj.2026.100927 (PMC13470313; doi:10.1016/j.xnsj.2026.100927)
Supplement: Supplementary file 1 [file mmc1.docx]

## **Appendix A. Patient demographics**

**Table A-I:** Patient demographics and clinical data of all 67 patients included in at least one of the three machine learning models. MAP: Mean arterial pressure, C: Cervical, T: Thoracic, L: Lumbar, S: Sacral.

| **Patient** | **Age (Years)** | **Mean intraoperative MAP (mmHg)** | **Reason for surgery** | **Preoperative neurological deficits** | **Three months postoperative neurological outcome** |
| --- | --- | --- | --- | --- | --- |
| **1** | 85 | 78.39 | Extirpation extramedullary tumor C3-C5 | Yes | Neurological improvement |
| **2** | 42 | 94.97 | Extramedullary tumor L4 | No | Neurologically intact |
| **3** | 65 | 81.81 | Ll-L2 intramedullary tumor | Yes | Neurological improvement |
| **4** | 42 | 85.10 | Tumor L2 intradural | No | Neurologically intact |
| **5** | 45 | 69.32 | Untethering (laminectomy L5) | Yes | Neurological improvement |
| **6** | 43 | 99.11 | Intradural extramedullary tumor T4 | Yes | Neurologically stable deficits |
| **7** | 46 | 99.30 | Resection intradural tumor C7 | No | Neurologically intact |
| **8** | 70 | 76.67 | Thoracic tumor | Yes | Neurological improvement |
| **9** | 69 | 72.24 | Intradural extramedullary tumor T3 | No | Neurological improvement |
| **10** | 76 | 88.85 | Tumor T11-T12 | Yes | Neurological improvement |
| **11** | 81 | 79.59 | Tumor extirpation L4-L5 | No | Neurologically stable deficits |
| **12** | 60 | 98.24 | Extirpation intradural tumor T1-T2 | Yes | Neurological improvement |
| **13** | 54 | 87.25 | Tumor extirpation C3-C4 | Yes | Neurological improvement |
| **14** | 68 | 81.71 | Resection intradural tumor T4-5 | Yes | Neurologically stable deficits |
| **15** | 50 | 91.93 | Resection intradural tumor L1-L2 | Yes | Neurological improvement |
| **16** | 57 | 107.78 | Resection intradural tumor T7-T8 | Yes | Neurological improvement |
| **17** | 72 | 73.30 | Fixation T10-S1 | Yes | Neurological improvement |
| **18** | 41 | 81.81 | Kyphosis correction T2-10 | Yes | Neurological improvement |
| **19** | 64 | 78.95 | Tumor extirpation L2 | No | Neurologically intact |
| **20** | 51 | 78.98 | Biopsy spinal cord tumor C5-6 | Yes | Neurological improvement |
| **21** | 35 | 70.45 | Scoliosis correction T1-L3 | No | Neurologically intact |
| **22** | 77 | 80.26 | Extramedullary intradural tumor T4 | Yes | Neurological improvement |
| **23** | 58 | 72.85 | Extirpation intraspinal osteophyte T12 | Yes | Neurologically stable deficits |
| **24** | 54 | 77.34 | Cystic lung metastasis L3-L4 | Yes | Neurological improvement |
| **25** | 45 | 88.67 | Ependymoma T8-T10 | Yes | Neurologically stable deficits |
| **26** | 79 | 93.73 | Vertebral Fusion Surgery T8-L3 | No | Neurologically intact |
| **27** | 26 | 76.36 | Extramedullary tumor T3-T4 | No | Neurologically intact |
| **28** | 68 | 92.06 | Vertebral Fusion Surgery T10-S1 | Yes | Neurological improvement |
| **29** | 53 | 69.05 | Intradural tumor L1-L2 & sacral | No | Neurologically intact |
| **30** | 19 | 77.76 | Resection schwannoma T11 | Yes | Neurological improvement |
| **31** | 50 | 91.44 | Resection intradural tumor L4 | No | Neurologically intact |
| **32** | 68 | 82.55 | Intradural tumor Th10 | Yes | Neurological improvement |
| **33** | 77 | 87.22 | Resection intradural tumor L5-S1 | Yes | Neurological improvement |
| **34** | 59 | 87.37 | Scoliosis correction Th6-S1 | Yes | Neurological improvement |
| **Patient** | **Age (Years)** | **Mean intraoperative MAP (mmHg)** | **Reason for surgery** | **Preoperative neurological deficits** | **Three months postoperative neurological outcome** |
| **35** | 25 | 72.70 | Resection intradural tumor C7 | No | Neurologically intact |
| **36** | 26 | 85.73 | Resection intradural tumor Th12 | No | Neurologically intact |
| **37** | 24 | 85.12 | Unilateral cordotomy low thoracic | Yes | Neurological improvement |
| **38** | 61 | 88.15 | Scoliosis correction Th12-L4 | Yes | Neurological improvement |
| **39** | 38 | 70.40 | Untethering lipoma cauda equina | Yes | Neurological improvement |
| **40** | 37 | 72.16 | Intramedullary cyst C4-C7 | No | Neurologically intact |
| **41** | 69 | 85.61 | Intradural tumor C7-Th1 | No | Neurologically intact |
| **42** | 73 | 92.47 | Biopsy intramedullary tumor T5-Th7 | Yes | Neurologically stable deficits |
| **43** | 66 | 83.16 | Intradural tumor conus medullaris | Yes | Neurological improvement |
| **44** | 75 | 81.81 | Extirpation tumor Th11-12 | No | Neurologically intact |
| **45** | 53 | 87.15 | Corpectomy C5 and C6 | Yes | Neurological improvement |
| **46** | 75 | 87.175 | Tumor extirpation Th5 | Yes | Neurological improvement |
| **47** | 74 | 82.54 | Tumor extirpation Th9-Th10 | No | Neurologically intact |
| **48** | 69 | 92.18 | Tumor extirpation Th1-Th2 | Yes | Neurological improvement |
| **49** | 24 | 83.64 | Tumor extirpation L2-L3 | Yes | Neurological improvement |
| **50** | 41 | 65.07 | Tumor L3-L4 | No | Neurologically intact |
| **51** | 56 | 74.97 | Laminectomy C3-C7 | Yes | Neurologically stable deficits |
| **52** | 57 | 76.79 | Laminectomy and lateral fixation C4–C6 | Yes | Neurological improvement |
| **53** | 87 | 102.00 | Extirpation meningioma Th10-Th11 | Yes | Neurological improvement |
| **54** | 53 | 80.54 | Decompression Th2-Th5 & L3-L5 | Yes | Neurologically stable deficits |
| **55** | 69 | 78.66 | Scoliosis correction Th11-ilium | No | Neurologically intact |
| **56** | 77 | 71.43 | Resection intradural tumor C6-C7 | No | Neurological improvement |
| **57** | 66 | 78.73 | Extirpation tumor Th9-Th10 | Yes | Neurological improvement |
| **58** | 59 | 76.38 | Scoliosis correction Th10-S1 | Yes | Neurological improvement |
| **59** | 49 | 66.21 | Resection conus medullaris Th11-Th12 | Yes | Neurologically stable deficits |
| **60** | 54 | 79.48 | Thoracic kyphosis | No | Neurologically intact |
| **61** | 61 | 101.52 | Scoliosis correction Th10-ilium | No | Neurologically intact |
| **62** | 85 | 83.65 | Extirpation meningiomas Th5 and Th8 | Yes | Neurological improvement |
| **63** | 84 | 135.92 | Laminectomy C7-Th1 | Yes | Neurological improvement |
| **64** | 50 | 71.64 | Untethering spinal cord | Yes | Neurological improvement |
| **65** | 39 | 61.66 | Untethering spinal cord | Yes | Neurological improvement |
| **66** | 58 | 89.75 | Removal of hemangioblastoma Th8-9 | Yes | Neurologically stable deficits |
| **67** | 71 | 92.88 | Ependymoma C3 | Yes | Neurological improvement |

## **Appendix B. Sensitivity analysis MEP-SSEP model**

B

A

C

**Figure B-I:** Performance plots of the MEP-SSEP model trained with the XGBoost classifier without the feature Preoperative neurological deficits. A: ROC curves for each individual class, B: precision-recall curves for each individual class, C Feature importance plot with the top seven features. ROC: Receiving operator characteristic, MEP: Motor evoked potential, SSEP: Somatosensory evoked potential, XGBoost: Extreme gradient boosting
